# Impact of the gut microbiota and associated metabolites on cardiometabolic traits, chronic diseases and human longevity: a Mendelian randomization study

**DOI:** 10.1186/s12967-022-03799-5

**Published:** 2023-01-30

**Authors:** Eloi Gagnon, Patricia L. Mitchell, Hasanga D. Manikpurage, Erik Abner, Nele Taba, Tõnu Esko, Nooshin Ghodsian, Sébastien Thériault, Patrick Mathieu, Benoit J. Arsenault

**Affiliations:** 1 Centre de Recherche de L’Institut Universitaire de Cardiologie et de Pneumologie de Québec, Y-3106, Pavillon Marguerite D’Youville, 2725 Chemin Ste-Foy, Québec, (QC) G1V 4G5 Canada; 2 Estonian Genome Center, Institute of Genomics, University of Tartu, Riia 23B, 51010 Tartu, Estonia; 3 Institute of Molecular and Cell Biology, University of Tartu, Riia 23, 51010 Tartu, Estonia; 4 Department of Molecular Biology, Medical Biochemistry and Pathology, Faculty of Medicine, Université Laval, Québec, (QC) Canada; 5 Department of Surgery, Faculty of Medicine, Université Laval, Québec, (QC) Canada; 6 Department of Medicine, Faculty of Medicine, Université Laval, Québec, (QC) Canada

## Abstract

Features of the gut microbiota have been associated with several chronic diseases and longevity in preclinical models as well as in observational studies. Whether these relations underlie causal effects in humans remains to be established. We aimed to determine whether the gut microbiota influences cardiometabolic traits as well as the risk of chronic diseases and human longevity using a comprehensive 2-Sample Mendelian randomization approach. We included as exposures 10 gut-associated metabolites and pathways and 57 microbial taxa abundance. We included as outcomes nine cardiometabolic traits (fasting glucose, fasting insulin, systolic blood pressure, diastolic blood pressure, HDL cholesterol, LDL cholesterol, triglycerides, estimated glomerular filtration rate, body mass index [BMI]), eight chronic diseases previously linked with the gut microbiota in observational studies (Alzheimer’s disease, depression, type 2 diabetes, non-alcoholic fatty liver disease, coronary artery disease (CAD), stroke, osteoporosis and chronic kidney disease), as well as parental lifespan and longevity. We found 7 associations with evidence of causality before and after sensitivity analyses, but not after multiple testing correction (1198 tests). Most effect sizes (4/7) were small. The two largest exposure-outcome effects were markedly attenuated towards the null upon inclusion of BMI or alcohol intake frequency in multivariable MR analyses. While finding robust genetic instruments for microbiota features is challenging hence potentially inflating type 2 errors, these results do not support a large causal impact of human gut microbita features on cardiometabolic traits, chronic diseases or longevity. These results also suggest that the previously documented associations between gut microbiota and human health outcomes may not always underly causal relations.

**Supplementary Information:**

The online version contains supplementary material available at 10.1186/s12967-022-03799-5.

## Introduction

The human gut microbiota is the microbial symbiotic organ residing in the gut. It is involved in key metabolic and immunological processes including host immunity, food digestion, intestinal endocrine function and intestinal permeability [47]. Several observational studies revealed that the gut microbiota is associated with a wide range of cardiometabolic risk factors and human diseases [32]. The systemic effects of the gut microbiota is partly mediated through its by-products. These microbial metabolites can reach the peripheral circulation via the portal vein [6], or diffuse readily and be taken up by the gut mucosa [24], where they can reach organs and act as substrates or signalling molecules. This bidirectional crosstalk between the gut microbiota and different organs occurs via the gut-liver axis [2], the gut-brain axis [15], the gut-bone axis [66], the gut-kidney axis [22], the gut-lung axis [48] and the gut-heart axis [4]. Specific classes of microbiota-derived metabolites, notably short-chain fatty acids [19], branched-chain amino acids [3], trimethylamine N-oxide [58], and derivatives of tryptophan [5] have been implicated in the pathogenesis of metabolic disorders [1], lifespan [72], neurological and cardiovascular diseases [49].

Over the past few years, the gut microbiota emerged as a therapeutic target of great interest to prevent and/or treat chronic diseases and improve human lifespan and healthspan. An overwhelming amount of supportive evidence from preclinical models contributed to the widely accepted view that a large number of diseases and pathological processes could be influenced by the microbiome, from early metabolic perturbations to full-blown diseases and premature mortality. Fecal transplantation studies in rodents have provided promising results for the treatment of obesity [51], type 2 diabetes (T2D) [71], depression and chronic stress [42], liver injury [44], myocarditis [33] and aging [16]. Human microbiota-associated (HMA) studies, consisting of the transplantation of feces from human patients into germ-free mice while control mice receive feces from healthy humans, further supported these associations. A systematic review conducted in 2019 on the HMA method to study the impact of the microbiota on chronic diseases reported that 95% of such studies (36/38) concluded that fecal transplantation from a sick human donor resulted in at least one worsened symptom compared to healthy controls [69].

This finding was deemed “implausible” by the authors of this systematic review [69]. According to Walter et al., in the vast majority of cases, these studies lacked adequate replication and they had statistical and methodological flaws that artificially inflated the odds of obtaining positive findings [69]. Together with the “file-drawer effect” (whereby positive studies are more likely to be published compared to negative studies), these caveats may distort the odds of translating findings from preclinical models into microbiota-targeting therapies to prevent or treat human diseases. Observational studies in humans with various diseases have identified relevant differences in intestinal microbiota composition [59]. However, they are subject to biases such as reverse causality and confounding (through unmeasured confounders) and cannot, by design, assess causality. Obesity, pharmacotherapy, diet, alcohol intake and many other factors appear to be important confounders in the microbiota-health relationships [67]. Given these limitations, Walter et al. suggested that novel and innovative methods such as Mendelian randomization (MR) should be used to investigate the causal role of the gut microbiota in human disease etiology.

MR is an epidemiological approach that mitigates many of the biases of observational studies such as reverse causality or confounding. Under specific assumptions, it has the potential to evaluate potential causal effects between multiple exposures (gut microbiota features) and outcomes (cardiometabolic traits, chronic diseases and human longevity). Briefly, MR uses genetic variants strongly associated with an exposure (gut microbiota features) to infer causality with an outcome (cardiometabolic traits, chronic diseases or human longevity). Twin studies have shown that heritability of the abundance of different bacterial taxa is on average 20%, although some variation exists between taxa [26, 27]. This is consistent with the view that genes play a non-negligible role in determining gut microbiota composition, making MR a valuable tool to assess the potential causal role of the gut microbiota in human diseases.

Here, we used a 2-sample MR study design to investigate the potential causal links between gut microbiota features and nine cardiometabolic traits (fasting glucose, fasting insulin, diastolic blood, systolic blood pressure, HDL cholesterol, LDL cholesterol, triglycerides, estimated glomerular filtration rate, body mass index [BMI]) eight chronic disease outcomes encompassing different body systems (coronary artery disease [CAD], T2D, ischemic stroke [IS], nonalcoholic fatty liver disease [NAFLD], chronic kidney disease [CKD], osteoporosis, Alzheimer disease [AD] and depression), and human lifespan (as defined by parental lifespan and living beyond the 90^th^ percentile). In this large-scale MR study, we first investigated the potential causal effect of micriobiota associated metabolites on diseases, metabolic risk factors and lifespan. Second, we leveraged summary statistics from two large genome-wide association studies (GWAS) of gut microbiota abundance to investigate the causal effect of genetically predicted taxa abundance on chronic diseases and human longevity.

## Results

**Fig. 1:**
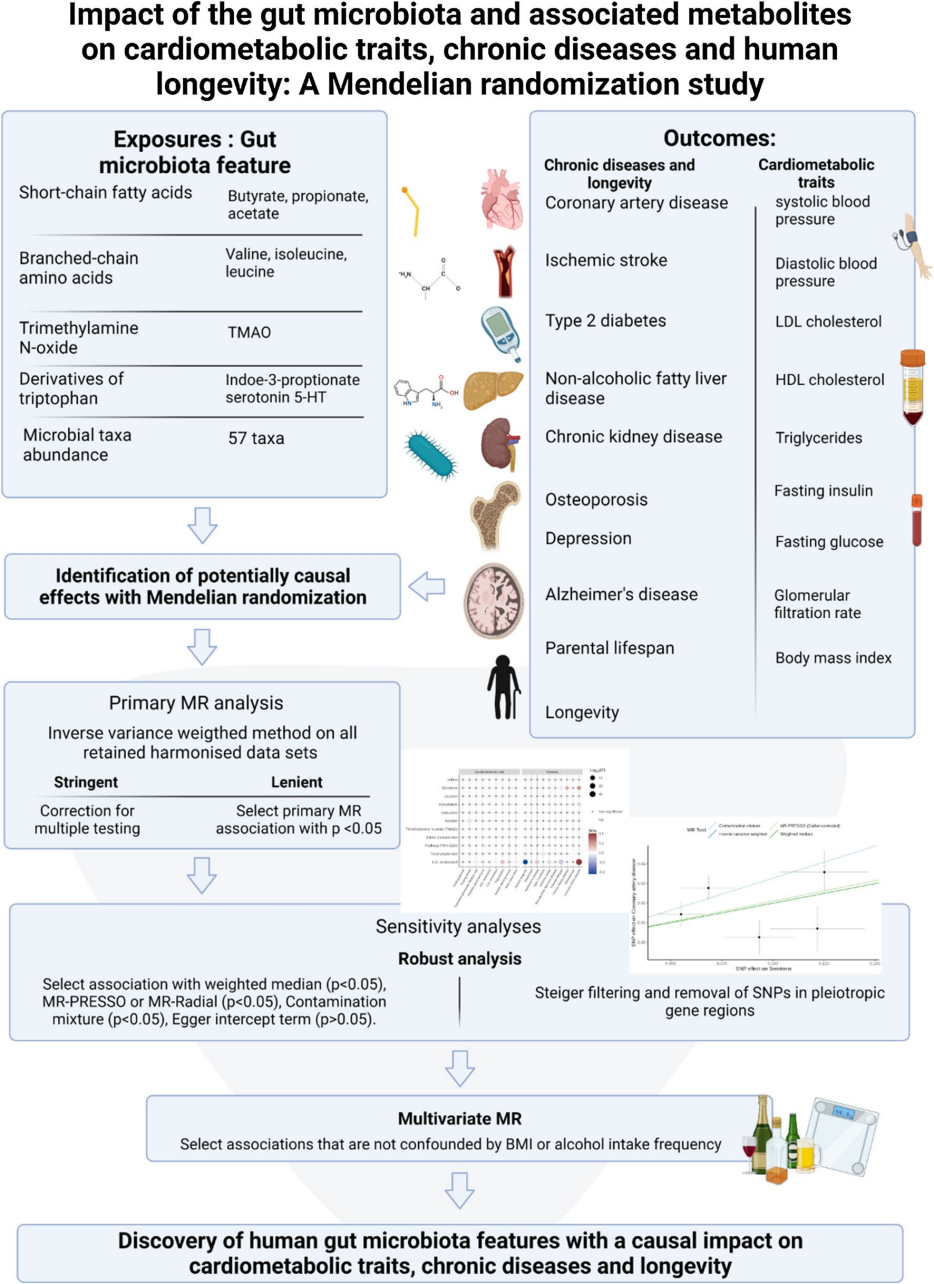
Overview of the Mendelian randomization framework used to investigate the causal effect of gut microbiome features (blood and gut-derived metabolites and microbial taxa abundance) on cardiometabolic traits, chronic diseases and human longevity

The conceptual framework of this MR analysis as well as the datasets used to derive the study exposures and outcomes are presented in Fig. 1 and Additional file 1: Table S1. Briefly, the objective of this MR analysis was to test the hypothesis that the gut microbiome causally impacts chronic diseases and longevity and to provide estimates for each exposure/outcome association. We performed two sample MR on microbiota features as exposures and relevant outcomes of cardiometabolic risk factors, chronic diseases and human longevity. We used publicly available genome-wide association study (GWAS) summary statistics to extract 67 traits related to the microbiome including 10 fecal and plasma metabolites associated with the gut microbiota, microbial abundance of 57 taxa partly under genetic control, nine cardiometabolic risk factors and 10 disease-related outcomes and human longevity (see Methods). The ten metabolites were selected based on the existence of taxa that metabolize them and based on their previous association with chronic diseases, as described in Additional file 1: Table S2. Analyses were restricted to participants from European ancestry except for a fraction of participants included in the study of microbial abundance (MiBioGen) consortium and CAD (CARDIoGRAMplusC4D). Samples from exposures and outcomes overlapped to a minor extent and at different degrees depending of data sources. We selected only exposures that had at least three independent (r2 < 0.01) genetic instruments at minimum *p*-value < 1e-5 (the threshold differed between data source depending on the availability of genetic instruments [Additional file 1: Table S3]) with mean F statistics  > 10, resulting in 67 microbiota-related exposures available for MR. These criteria were chosen to minimize weak instrument bias and allow the use of sensitivity analysis to assess the validity of the MR assumptions. The harmonized dataset of the associations between genetic variant and exposure, and between genetic variant and outcome is presented in Additional file 1: Table S4.

### Effect of gut microbiome-related metabolites on chronic diseases and longevity

**Fig. 2:**
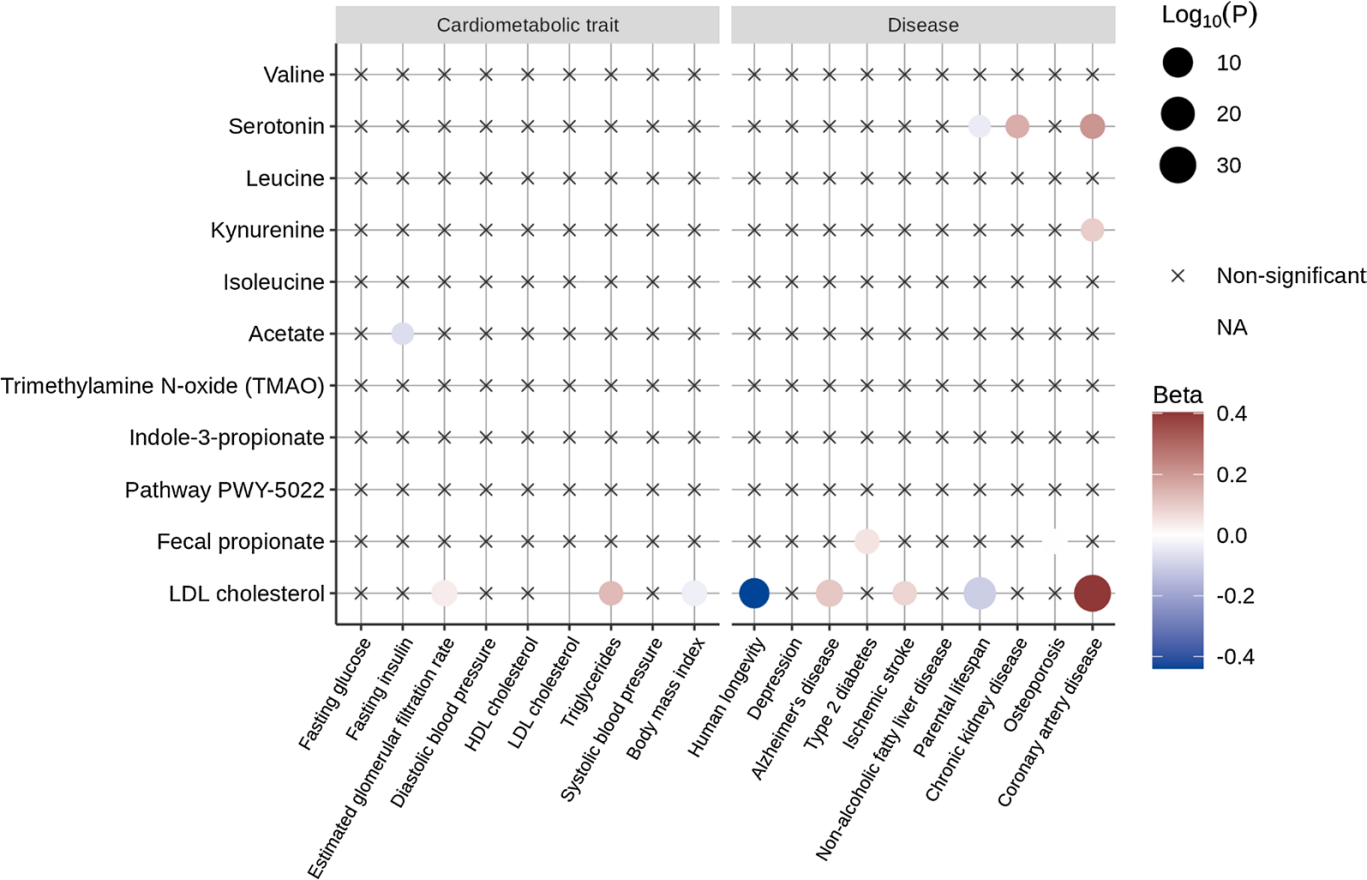
Balloon plot of the association of microbial fecal metabolites, microbial pathway and plasma metabolites with all 19 health outcomes. LDL cholesterol is included as positive control. Non-available (NA) associations stem from a lack of overlapping SNPs or proxies between exposure and outcome data resulting in fewer than three genetic instruments in the harmonized data set. Associations at *P*-value  > 0.05 are depicted with crosses. For readability, the effect of LDL on LDL was forced to be non-available

We first sought to determine whether 10 blood metabolites associated with the gut microbiota and fecal microbial metabolites or their functional pathways could influence cardiometabolic risk factors, chronic diseases and longevity. We selected genetic instruments for short-chain fatty acids such as fecal propionate, a gut metabolite linked with T2D in a recent MR study, from a GWAS of 898 participants [56] and acetate [36]. We included the microbial pathway involved in 4-aminobutanoate (GABA) degradation (PWY-5022 pathway) acting as a proxy for butyrate production by the gut [56]. We also included trimethylamine N-oxide [54], branched chain amino acids (leucine, isoleucine and valine) [46] and derivatives of tryptophan produced by the gut microbiota, namely indole 3-propionate [54], serotonin and kynurenin [46]. We performed IVW-MR for each of the health outcomes under study (Fig. 2). A total of 190 exposure-outcome associations were tested. The mean absolute effect size was 0.04, meaning that a 1-SD deviation increase in a microbiota associated metabolites would only increase by 0.04 SD a risk factor or by ~ 4% the risk of a disease. Seven associations passed a nominal p-value significance threshold of 0.05 (including the propionate-T2D association) but none of the gut microbiota metabolites were associated with chronic diseases and longevity after multiple testing correction. Figure 2 also reports the association of LDL cholesterol, used as positive control, with the outcomes of interest. As expected, each SD increase in LDL cholesterol was positively associated with cardiovascular diseases (OR = 1.5 95% CI 1.4–1.6, *P* = 1.4e-31) and negatively associated with human longevity (OR = 0.64 95% CI 0.56–0.74, *P* = 8.4e-11). Altogether, this analysis identified some blood and gut-derived metabolites that may be associated with cardiometabolic traits, chronic diseases and human longevity but their effect sizes were small. Spurious associations also cannot entirely be ruled out since none of the metabolites were associated with outcomes of interest after correction for multiple testing.

### Effect of gut microbial abundance on chronic diseases and longevity

**Fig. 3:**
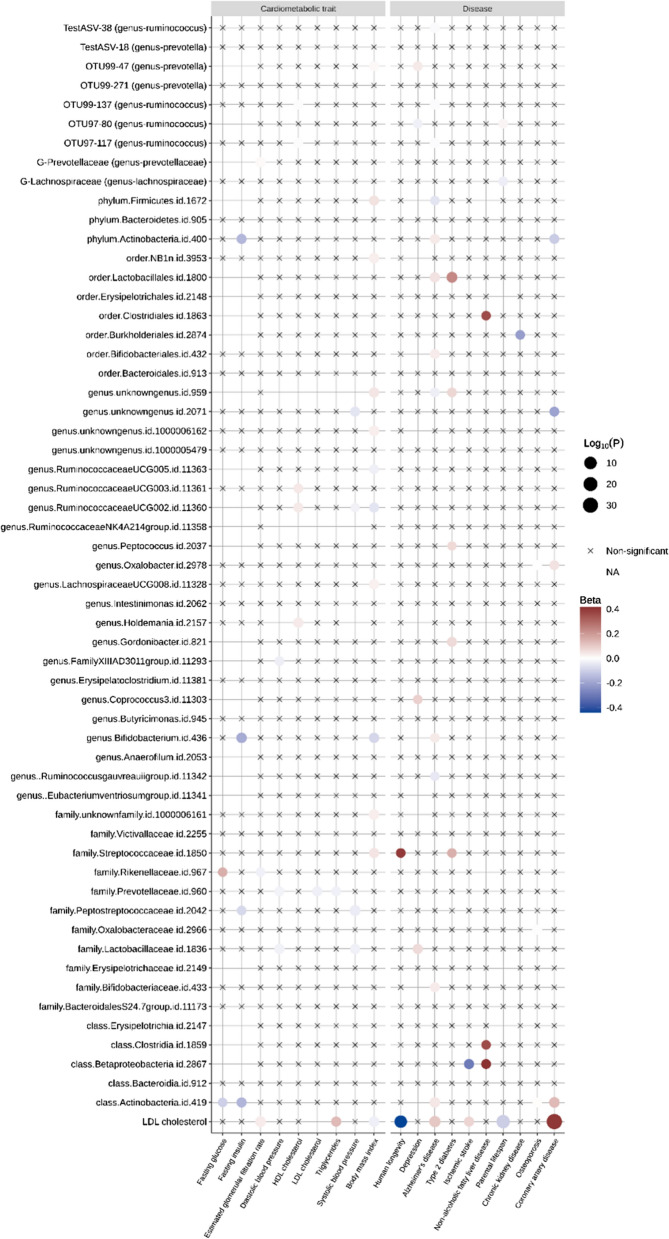
Balloon plot of the association of gut microbial taxa abundance with all 19 health outcomes. LDL cholesterol is included as positive control. Non-available (NA) associations stem from a lack of overlapping SNPs or proxies between exposure and outcome data resulting in fewer than three genetic instruments in the harmonized data set. Associations at *P*-value  > 0.05 are depicted with crosses. For readability, the effect of LDL on LDL was forced to be non-available

We explored the impact of different taxa abundance on health-related outcomes. We included available genetic information on 57 microbial taxa abundance from the two recent GWAS studies of Kurilshikov et al., and Rühlemann et al. [40, 55]. We then performed IVW-MR on each of the 19 health outcomes (Fig. 3). Similar to microbiota associated metabolites, the mean absolute effect size was 0.04. Out of 1008 exposure-outcome tests, 69 passed a significance threshold of 0.05, but no association remained after Benjamini-Hochberg correction for multiple testing. Altogether, this analysis identified some microbes that may be associated with cardiometabolic traits, chronic diseases and human longevity but, as observed with blood and gut-derived metabolites, reported effect sizes were small.

### Exploration of promising findings and tests for pleiotropy

**Fig. 4:**
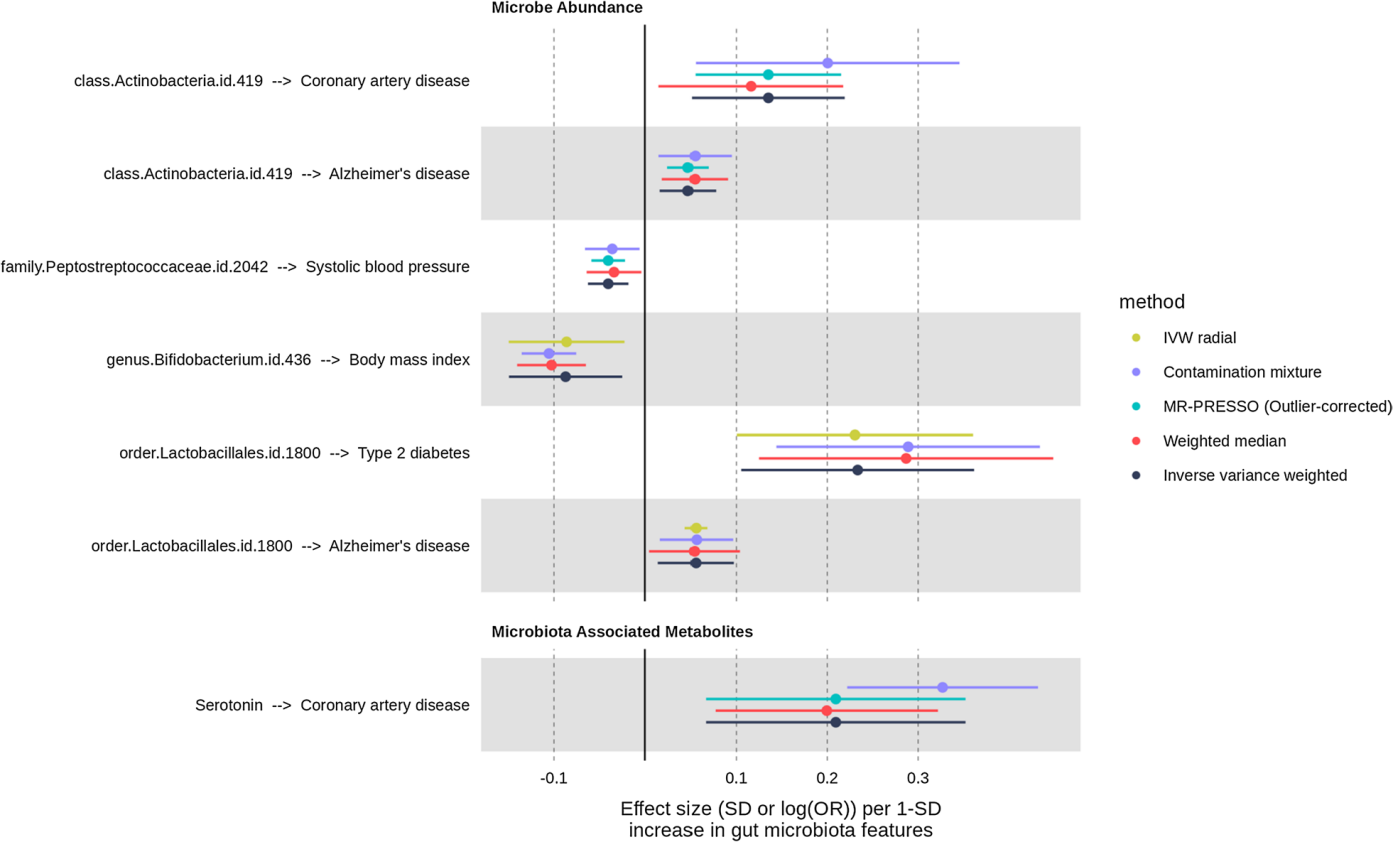
Forest plot of the associations that were consistent across robust MR analyses. Dichotomous traits are reported on a log(OR) scale. Continuous are reported on 1-SD scale. Dots depicts the point estimate. Horizontal bars depicts 95% confidence interval (CI)

One of the key assumptions underlying MR is that genetic instruments affecting gut microbiota features do not affect diseases or longevity by other mechanisms than the one associated with gut microbiota features (Davies, Holmes, and Davey Smith 2018). This phenomenon is known as horizontal pleiotropy [43]. We performed robust MR analysis on all 76 potentially causal relationships (primary analysis *P*-value  < 0.05) to estimate the robustness of our primary causal estimates to pleiotropy. We used four different methods that make different assumptions about the nature of the underlying pleiotropy: the weighted median, the MR-PRESSO or when it could not be performed the MR-Radial and the contamination mixture approaches. Consistency across the estimates of the methods provides support to causality. The MR-Egger intercept was also used to assess robustness to horizontal pleiotropy. We removed the MR results that were not supported by robust MR analyses (weighted median method *P* > 0.05, MR-Egger intercept *P* < 0.05, MR PRESSO outliers-adjusted test or MR-Radial test (*P* > 0.05), contamination mixture (*P* > 0.05). Of the 76 associations tested, 7 remained as they were consistent with a true causal effect unlikely to be confounded by pleiotropy (Fig. 4 and Additional file 1: Table S6). Genetic instruments of these associations were located outside the ABO, HLA and APOE gene window (± 1 Mb), which may represent genetic regions potentially causing pleiotropy and hence were less likely to be pleiotropic. Genetic instruments did not display evidence of reverse causality as indicated by Steiger filtering tests. Most (4/7) causal effects were low (abs(b)  < 0.1). Notably, the causal effect of circulating serotonin levels on CAD and the causal effect of the order *lactobacillales* on T2D were strong (OR = 1.23 95% CI 1.07–1.42, *P* = 4.0e-03) and (1.26 95% CI 1.11–1.44, *P* = 3.6e-04) respectively. This analysis identified some gut microbiota associated features that may be worth exploring in further studies.

### Exploration of BMI and alcohol intake as potential confounding factors

**Fig. 5:**
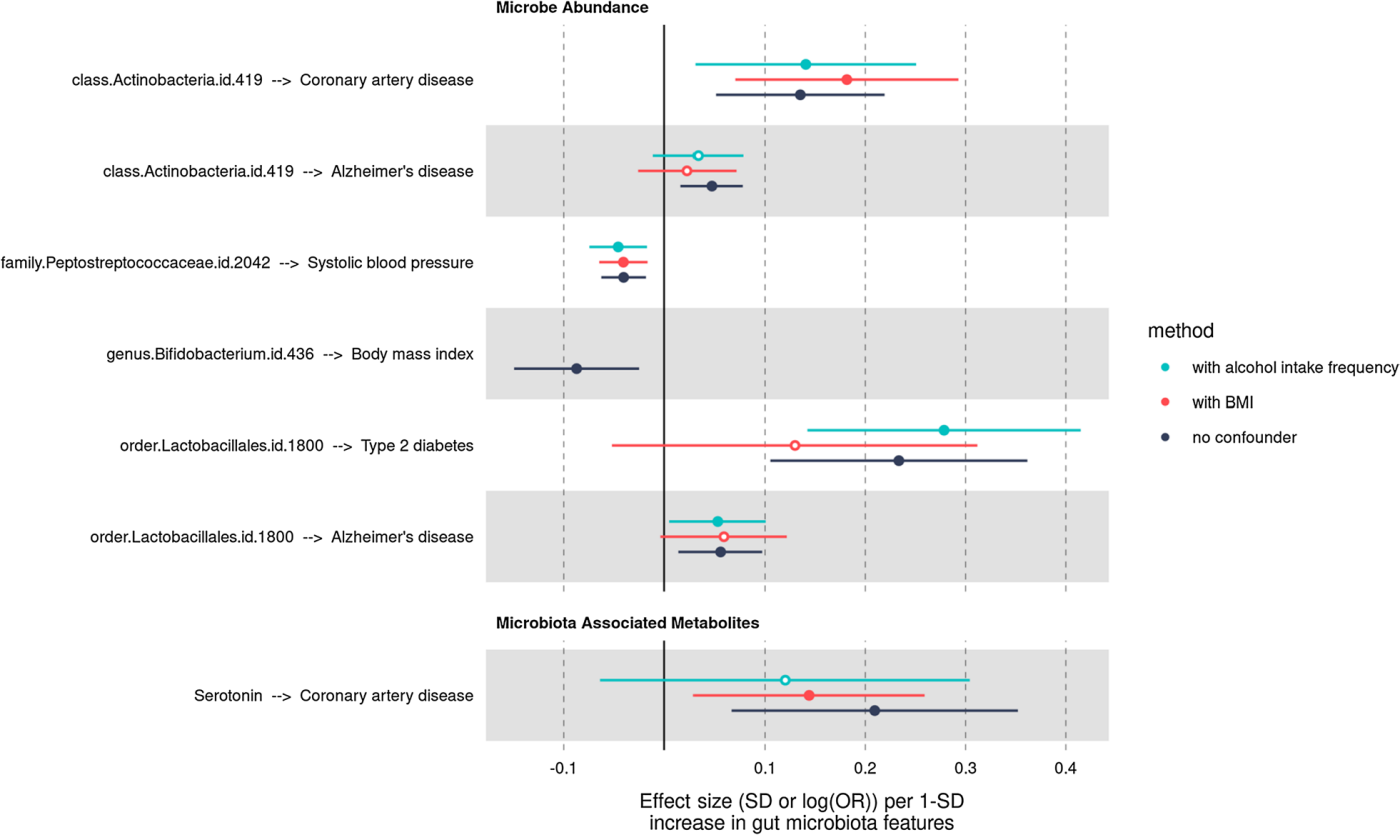
IVW-MR results before and after correcting for BMI and alcohol intake frequency using multivariable MR framework

Obesity and alcohol intake frequency were recently identified as major confounding factors in microbiome-disease associations [67]. We performed multivariable MR on the 7 promising associations to determine if the causal effects were robust to the inclusion of obesity and alcohol intake frequency. This analysis provided evidence that the two aforementioned largest reported associations might, to a certain extent, be confounded by BMI or alcohol consumption (Fig. 5 and Additional file 1: Table S7). For example, the causal effect of serotonin plasma level on CAD attenuated towards the null to (1.13 95% CI 0.94–1.36, *P* = 2.0e-01) upon inclusion of alcohol intake frequency as covariate and the causal effect of the order Lactobacillales on type 2 diabetes risk attenuated towards the null to (OR = 1.14 95% CI 0.95–1.37, *P* = 1.6e-01) upon inclusion as BMI as covariate.

## Discussion

In order to determine whether previously reported studies using preclinical models or observational study designs in humans were consistent with causal effects, we assessed the roles of a wide range of microbial factors and nine cardiometabolic risk factors, eight chronic diseases as well as human longevity using MR. We found 7 associations with evidence of causality before and after sensitivity analyses, but not after multiple testing correction.Most effect sizes were small. The causal effect of serotonin levels on CAD and of the Lactobacillales order abundance on T2D were strong, but their effect substantially decreased upon inclusion of BMI and alcohol intake frequency in multivariable MR analyses. Altogether, results of this study suggest that previously reported associations between the human gut microbiome and human disease might have been due to biases such as reverse causality or confounding and that the impact of the gut microbiota on cardiometabolic traits, chronic diseases and human longevity may not be as prominent as previously suggested.

### Comparisons with other studies

Our results generally contrast with those from previous observational studies. Microbial metabolites have been associated with health and disease such as neurological disease, NAFLD, cardiovascular disease, survival and T2D (Agus, Clément, and Sokol 2020; [49, 72]. In a prospective study of apparently healthy participants with eight-year follow-up, elevated plasma TMAO (4th quartile vs. 1st quartile) predicted CAD even after adjustments for traditional risk factors (adjusted odds ratio (OR) 1.58 [95% confidence interval (CI) 1.21–2.06], *P* < 0.001) [62]. This result was similar in another study cohort [29]. In mice, increasing through dietary supplementation the level of TMAO accelerated atherosclerosis [39]. By contrast, our MR analysis does not support causality of TMAO on CAD (0.99 95% CI 0.98–1, *P* = 9.9e-02), despite precisely estimating the effect size, as previously suggested in another MR investigation on CAD [34]. Dietary factors could arguably act as confounding factors, since meat intake increases TMAO levels [39].

Several differences in the microbial composition of diseased and healthy individuals have been identified, but causality remains to be elucidated. RCT of fecal microbiome transfer (FMT) in humans are currently employed to establish causality between microbiome and health, but few have been attempted, and even fewer have been conclusive [20, 50]. To date, most successful randomized control study of FMT on humans has been applied to the treatment of recurrent or refractory *Clostridioides difficile* infections [73] and some to ulcerative colitis [17]. Mice FMTs are a valuable exploratory tool, but inference to human subjects is hazardous. Particularly, a substantial proportion of species in the human gut are not present in mice [37]. For example, several FMT in mice from lean to obese mice resulted in improved cardiometabolic profile [41, 76], but these findings failed to replicate in humans. A systematic review of all three randomized placebo-controlled studies to treat obesity published to date found no impact of FMT on obesity, fasting plasma glucose, hepatic insulin sensitivity, or cholesterol markers across all included studies [75]. For human observational studies, multiple confounding factors could create spurious correlation between microbiome and chronic diseases, including antibiotic use, age, sex, diet, geography, BMI and alcohol intake [38]. Moreover, alteration of the gut microbiota could potentially be a consequence of disease states rather than a causal factor [14].

Altogether, the large proportion of null findings (i.e., human gut-related traits may not cause chronic disease) is in line with recent literature showing an overwhelming positive publication bias in the microbiome literature [69]. Publication bias can occur if studies are not adequately corrected for multiple testing and can be identified with attempt and failure to replicate (i-e winners curse bias) [57]. For example, it was originally published that individuals with obesity were more likely to have lower bacterial diversity and relative abundances of the phylum Bacteroidetes [63], but this result failed to replicate in 9 independent cohorts [23, 61, 70]. This highlight the importance of triangulating with different methods such as MR to address a causal research question.

### Strengths and limitations

An important strength of this study is the use of the MR design with the largest publicly available GWAS datasets. Because alleles are randomly assigned and fixed at conception, biases due to confounding and reverse causality are mitigated in an MR analysis [18]. A further strength is that sample was mostly restricted to individuals of European ancestry to reduce bias due to population stratification. However, it also restricts the generalizability of the results to this ethnic group.

Our study, however, has limitations. Robust genetic instruments for microbial species are challenging to find. First, microbiome heterogeneity and interindividual variability are high, substantially reducing the statistical power of microbiome GWAS analyses. Second, the phenotype is distal from individual genes making it a complex polymorphic trait with many variants of small effect size which could be prone to pleiotropy. Lastly, the twin heritability for gut microbiota taxa abundance is only on average 20% [26, 27]. This heritability estimate represents the upper ceiling that variance explained by genetic instruments can attain, reducing power. These factors all contributed to the fact that the number of mbQTLs identified to date is rather modest. For these reasons, we used a less stringent p-value cut-off to include a greater number of genetic variants to allow the use of sensitivity analysis and increase power. However, a less stringent p-value cut-off has the trade-off to potentially increase the chance of including false-positive effect variants which induce biases. The most important bias it introduces, the winner’s curse bias, refers to the fact that the genetic hits in discovery samples are more likely to be false positive, adding noise to the analysis which will typically bias MR results towards the null. Second in importance, the weak instrument bias occurs when the variance explained by the instrument and the sample size are low [13]. In the two sample MR setting, it will bias towards the null [11]. Third in importance, invalid instruments are pleiotropic variants that affect the outcome via another pathway than the one going through the exposure. Since genetic variants are derived from the host, instruments for the microbiome as exposure have a high chance of being pleiotropic. In general, pleiotropy is more likely to bias estimates away from the null [60]. Therefore, our null results are generally more robust to horizontal pleiotropy. We minimized weak instrument biases by including only exposures with genetic instruments with an average F-statistic above 10. We minimized the propensity of the results to be biased by pleiotropic variants by systematically including sensitivity analyses.

The Steiger test we performed can be biased in the presence of a difference in measurement error between the exposure and the outcome [30]. Typically non-differential measurement error will decrease variance explained hence will bias the Steiger test towards lower measurement error to higher measurement error directionality [30]. Since microbiota features (our exposures) are arguably measured less precisely than the health phenotype (our outcomes), one can assume that Steiger filtering will be biased towards the reverse direction. This bias should have little impact on our results since no genetic instruments were removed by Steiger filtering.A second limitation is that the microbiome GWAS included in the current analysis did not target the entire 16S RNA gene, which greatly diminished their ability to achieve a sufficient taxonomic resolution to identify potential therapeutic targets. The meta-analysis by Ruhlemann targeted the V1-V2 subregion while the meta-analysis by Kurilshikov et al. included mostly the V4 subregion and to a lesser extent the V1-V3 subregion. Targeting only 16S subregions such as V4 leads to lower taxonomic resolution achieved compared to sequencing the full V1-V9 16S RNA gene [35]. Indeed, using a variable region as a surrogate for the entire 16S RNA gene only allows for the identification of taxa at the genus level or above [35]. Being confident at the genus level provides little to inform disease treatment. Indeed, within a high-level taxon such as a phylum, some species may have a positive correlation with a disease, but some neutral or negative. For example, in our study, the phylum Actinobacteria was potentially protective for CAD, while the subsequent level, the class Actinobacteria was a potential risk factor for the same disease. Employing third-generation technologies has the potential to allow the sequencing of the full 16S RNA gene in a high throughput manner, and improve taxonomic discrimination.

A third limitation is the difference in ancestry from data in the MiBioGen and CARDIoGRAMplusC4D consortium that could potentially violate the independence assumption through population stratification. Population stratification occurs in the advent that population subgroups have different disease rates or different distributions of continuous traits and have different frequencies of alleles. This phenomenon could violate the independence MR assumption. However, this bias is likely to be minimal as principal components were included and participants were mostly from European descent.

### Conclusions

Using MR, an approach less subject to reverse causality and confounding factors in comparison to traditional methods, we showed that several features of human gut microbioata including plasma metabolites and microbial taxa abundance had no evidence of causal effect on nine cardiometabolic traits, eight chronic diseases and human longevity. While finding robust genetic instruments for microbiota features is challenging potentially inflating type 2 errors, these results do not support large causal effects of the human gut microbiota and microbial metabolites on human chronic diseases and longevity. As the microbiome field matures, the use of larger microbiome GWAS study taking advantage of discriminatory potential of the full 16S RNA gene is warranted to fully elucidate the association of the human gut microbiota in the etiology of chronic diseases.

## Methods

### Study exposures (gut microbiota-derived metabolites)

We derived our ten gut microbiota-derived exposures of interest from five different publicly available data sources (Additional file 1: Table S1). We selected independent (r2 ≤ 0.01) SNPs (for all studies: *P*-value  < 1e-5; except Lotta et al.: *P*-value  < 1e-6) as genetic instruments. Genetic instruments for fecal propionate and PWY-5022 were obtained from a GWAS on 952 normoglycemic participants of the LifeLines-DEEP cohort, a population-based cohort from northern Netherlands (age ranges 18–84 years) [56]. Fecal propionate levels were measured by gas chromatography-mass spectrometry (GCMS). The functional pathway PWY-5022 was obtained with HUMAnN2 (v 0.4.0) [25] and MetaCyc metabolic-pathway database [64]. Genotyping was carried out with two Illumina arrays, HumanCytoSNP-12 BeadChip and ImmunoChip.

Genetic instruments for plasma TMAO and indole-3-propionate were extracted from a GWAS conducted in 2076 participants from European ancestry from the Framingham Heart Study (FHS) offspring cohort [54]. The FHS offspring cohort is a prospective community-based cohort from Framingham, Massachusetts, USA. Children of the spouse of the FHS study were recruited in 1971. Metabolites profiling was performed by liquid chromatography-mass spectrometry ^63^. Genotyping was conducted using the Affymetrix 500 K mapping array and the Affymetrix 50 K gene-focused MIP array. The participants all provided their informed consent and the study was approved by the Boston University Medical Center.

Genetic instruments for acetate were extracted from a meta-analysis of GWAS conducted on 10 European cohorts totalizing 24,925 individuals [36]. Human blood metabolites were quantified with quantitative high-throughput NMR metabolomics platform. Genetic instruments or plasma branched-chain amino acids (leucine, isoleucine and valine) and tryptophan derived metabolites kynurenine and serotonin were extracted from a meta-analysis of seven cohorts on up to 86,401 participants [46]. Human blood metabolites were quantified with quantitative high-throughput NMR metabolomics platform.

### Study exposures (gut-microbiata abundance)

We first identified 4 recent GWAS on gut microbe abundance with available summary statistics [40, 45, 53, 55]. We filtered all microbiota quantitative trait loci (mbQTLs) with *P*-value  < 1.0e-6 for all taxa abundance present in their analysis and kept only exposures with at least three shared mbQTLs with mean F statistics  > 10. The study of Lopera-Maya et al., and Qin et al., were removed as none of the exposures satisfied our criteria (≥ 3 independent mbQTLs at a *P*-value  < 1e-6, with mean F-statistics  > 10). In total, we derived our microbiota taxa abundance exposures of interest from two different publicly available data sources (Additional file 1: Table S1). Genetic instruments for bacterial taxon were extracted from a GWAS of bacterial taxon abundances of 8,956 German individuals from the PopGen (population-based cohort), the FoCus (population registry based), the KORA FF4 (population-based adult cohort initiated in 1984) and the SHIP cohort (longitudinal population-based cohort)^67^. Human Genotyping and fecal microbial 16S rRNA gene surveys were performed using multiple arrays. Additionally, other genetic instruments for bacterial taxon were extracted from a meta-analysis conducted by the MiBioGen consortium on 16S fecal microbiome data from 18,340 individuals (24 cohorts) [40]. All cohorts implemented the standardized 16S processing pipeline that uses SILVA as a reference database, with truncation of the taxonomic resolution of the database to genus level. Cohorts were Middle Eastern, East Asian, American Hispanic/Latin, African American and admixed, although the majority of the sample (more than 72%) came from European descent. Notably, The two studies shared the PopGen (n = 721), the FOCUS (n = 960), and the SHIP (n = 1901) cohorts for a total of 3582 individuals or approximately half the samplesize of the study by ruhlemann et al. For this reason, we only sourced genetic instruments in the study by Ruhlemann et al., for exposures that were absent from Kurilshikov.

### Study outcomes

We used publicly available GWAS summary statistics of the largest GWAS of nine cardiometabolic traits, eight chronic diseases, parental lifespan and longevity. Relevant information on the GWAS summary statistics are presented in Additional file 1: Table S1.

### Selection of genetic variants and variants harmonization

We first identified all SNPs associated with exposures. Summary parameters for genetic instrument selection can be found at Additional file 1: Table S7. These SNPs were then clumped using the 1000Genomes Project Phase 3 European LD reference panel to make sure instrumental variables were independent with a 10 Mb window and pairwise linkage disequilibrium (LD) r2 < 0.01. This step was implemented with the *gwwasvcf* package in R [21]. Instrument strength was quantified using the F-statistic [13], and the variance explained was quantified using the r^2^ [52]. Variant harmonization was performed by aligning the betas of different studies on the same effect allele with the *TwoSampleMR* package [31]. When a particular exposure SNP was not present in the outcome dataset, we used proxy SNPs instead (r2 > 0.8). We used the LD matrix of the 1000 Genomes Project-European sample of the Utah residents from North and West Europe. We kept only the results based on at least three independent shared SNPs with mean F statistics  > 10 to reduce weak instrument bias and allow for robust MR analyses.

### Primary Mendelian randomization analyses

We conducted primary MR analysis on each outcome and exposure association. As primary method for causal inference, we performed the IVW method with multiplicative random effects with a standard error correction for under dispersion as recommended by recent MR guidelines [10]. The IVW-MR combines the ratio estimates from each genetic instrument in a meta-analysis model by giving more weight to the ratio estimates with lower variance [12]. A total of 1198 primary analyses were performed: 67 exposures * 19 outcomes—75 exposures/outcomes with fewer than three overlapping genetic instruments or proxies. We applied a Benjamini Hochberg correction for multiple testing using a false discovery rate of 5% to reduce the propensity of false positive finding. For dichotomous traits, we transformed ORs and CIs to effect sizes and standard error when it was not already done. Lifespan, blood pressure, fasting glucose, fasting insulin, and glomerular filtration rate were reported in years, mmHG, log(pmol/L), log(pmol/L) and log(eGFR) respectively. For better interpretability and comparability, these summary statistics were transformed to a one standard deviation scale using the *sdY.est* function in the coloc package [68]. The other continuous variables were already inverse-rank normal transformed in the GWAS.

### Sensitivity analyses

For associations with IVW-MR *P*-value  < 0.05, we performed sensitivity analyses to estimate the robustness of our primary causal estimate. We used 5 different robust methods that make different assumptions about the nature of the underlying pleiotropy. As a general test of the presence of pleiotropy, we used the intercept term from MR-Egger regression), MR-Egger is similar to the IVW method except the regression model estimates an intercept [7]. An intercept significantly different from zero gives indication of pleiotropy. The contamination mixture provides consistent estimate under the plurality valid assumption [60]^59^. It assumes that not all genetic variants are valid IVs and runs a likelihood function to categorize genetic instruments as valid or invalid. As a general test of robustness of the IVW-MR estimates, we used the weighted median. The weighted median estimates an unbiased causal effect if the “majority valid” assumption is upheld, that is if up to 50% of the weights comes from variants that are valid IVs [8]. Finally, as a general test to the presence of outliers, we used the outlier-robust method MR-PRESSO, which is a simulation approach where genetic variants are removed based on their contributions to heterogeneity [65]. This method provides consistent estimates under the same assumptions as the IVW-MR method for the set of genetic variants that are not identified as outliers [60]. When the MR-PRESSO could not be performed because there was less than four instruments, we performed MR-Radial as an outlier robust test instead. The MR-Radial uses a simulation-based approach to detect and remove outlier variants to re-estimate the exposure-outcome relation [9].

As additional sensitivity analysis, we excluded variants from known pleiotropic gene regions and performed Steiger filtering. Because of their known association with pleiotropic pathways, we excluded from the analysis all SNPs of the *HLA*, *ABO* and *APOE* genetic regions. We also performed Steiger filtering to remove variants with evidence of a stronger association with the outcome than its association with the exposure. The Steiger test provides a p-value under the null hypothesis that the difference in variance explained is null [30].

Obesity and alcohol intake frequency were recently identified as major confounding factors in the gut-disease associations [67] because they are both to some extent associated with the health outcome under study while potentially simultaneously influencing microbiome composition. Although other confounding factors may exist, adding BMI and alcohol intake frequency are the most important predictors of microbiota composition and health and adding them as covariates in linear mixed-effect models reduced the numbers of spurious microbiome health associations [67]. To account for this, we performed multivariable MR as a sensitivity analysis to correct for measured confounders [28]. BMI and alcohol intake frequency GWAS from the UK biobank were obtained from publicly accessible source (Additional file 1: Table S1). Multivariable MR IVW estimates were computed using the *MendelianRandomization* package [74].

## Supplementary Information

**Additional file 1: Table S1.** Description of the datasets used. **Table S2.** Description of the metabolites selection rational. **Table S3.** Summary of the instrument selection criteria for each data source. LD R2, pvalue treshold and Fstatistics. **Table S4.** Harmonised dataset for each exposure outcome. **Table S5.** Primary MR results and associated statistics. **Table S6.** Robust MR results and other sensitivity analyses results. **Table S7.** Multivariable MR results and associated statistics.

## Data Availability

All data used in this study are in the public domain. Additional file 1: Table S1 describes the data used and relevant information to retrieve the summary statistics. Code was performed in the R V.4.0.0 computing environment using publicly accessible functions from the *TwoSampleMR* V.0.5.5 https://github.com/MRCIEU/TwoSampleMR, the *MendelianRandomization V.0.5.1* https://cran.rproject.org/web/packages/MendelianRandomization/index.html and the *data.table V.1.14.0* https://github.com/Rdatatable/data.table packages. The *tidyverse V.1.3.1* collection of R packages was also used. Code to reproduce the analysis can be found on https://github.com/gagelo01/Dysbiose_project.
